# Nomogram based on high-density lipoprotein cholesterol for the occurrence of preoperative deep vein thrombosis in patients with intertrochanteric femur fracture: a retrospective study

**DOI:** 10.1186/s13018-023-04497-8

**Published:** 2024-01-03

**Authors:** Wencai Li, He Ling, Rongbin Lu, Zhao Huang, Wei Su

**Affiliations:** 1 Dept. Orthopedics Trauma and Hand Surgery, The First Affiliated Hospital of Guangxi Medical University, No. 6 Shuangyong Road, Nanning, 530022 China; 2 Guangxi Collaborative Innovation Center for Biomedicine, Guangxi Medical University, Nanning, 530022 Guangxi China; 3 Huizhou Central People’s Hospital, Huizhou, 516001 Guangdong China

**Keywords:** Intertrochanteric femur fracture, Deep vein thrombosis, High-density lipoprotein cholesterol, Albumin, Lymphocytes, Nomogram

## Abstract

**Background:**

This study aims to develop a nomogram and forecast the incidence of DVT in individuals suffering from an intertrochanteric femur fracture.

**Method:**

This work created a nomogram using the R programming language and employed logistic regression to determine independent predicting features. An external validation dataset was used to validate the nomogram.

**Result:**

The findings demonstrated the independence of LYM (0.02[0.01–0.09], *p* < 0.001), ALB (0.83[0.74, 0.94], *p* = 0.002), and HDL-C (0.18[0.04, 0.71], *p* = 0.014). Good prediction performance with modest errors was shown by the nomogram in both the training and validation groups.

**Conclusion:**

In conclusion, the nomogram that was created using HDL-C, ALB, and LYM can assist medical professionals in determining the likelihood that DVT will occur.

## Introduction

Intertrochanteric femur fractures (IFF) have been more common in recent years [1]. With a 30-day mortality rate ranging from 1.0 to 6.5% and a one-year mortality rate considerably rising to 37.3%, IFF is the primary cause of death in the older population [2]. With its benefits of ease of use, affordability, and speed of examination, X-ray imaging is crucial in the diagnosis of IFF [3]. Patients with IFF need to stay in the hospital for an extended period of time, and they cannot return to their pre-fracture activities, joint function, or independent quality of life once they are discharged [4]. Patients with IFF have a much better prognosis after surgery [5], with two typical surgical techniques being intramedullary fixation using a proximal femoral nail system and extramedullary fixation using a plate system [6–8]. As the population ages, IFF is frequently associated with a number of serious side effects, such as deep vein thrombosis, nonunion of the fracture, and femoral head necrosis, which in extreme circumstances can be fatal [9]. Autonomy and physical function can be markedly enhanced by perioperative therapies [10].

Patients who have suffered lower limb fractures are frequently affected by deep vein thrombosis (DVT), which is linked to decreased blood flow velocity, decreased limb activity, and post-fracture limb oedema [11]. DVT incidence rates in trauma fracture patients range from 13.5 to 33.7%, according to published research [12]. Vascular colour Doppler ultrasonography is the main tool used to diagnose DVT, yet early on, patients with intertrochanteric fractures may find it difficult to cooperate [13, 14]. Pulmonary embolism is one potentially fatal outcome of DVT [15]. Early pharmacological and mechanical prophylaxis for DVT has been recommended by prior research; nevertheless, pharmacological prophylaxis has a risk of increased bleeding and brain haemorrhage because it impairs coagulation function [16]. For patients to receive the most benefit, early and precise prediction of DVT incidence is therefore essential, as it can substantially assist doctors in making early decisions [17].

The nomogram, one of the new prediction models, is a useful tool to help physicians because of its ease of use and friendliness. Nomograms are being created and validated by an increasing number of academics [18]. The necessity of creating a nomogram for DVT has been highlighted by the clinical consensus on early preventative intervention for DVT [19]. Jiabao Jiang and colleagues [20] used blood indicators to create a nomogram model for preoperative venous thrombosis in the calf muscles of older patients who had hip fractures. Using coagulation markers, Dongcheng Xu et al. [21] created a nomogram model for postoperative DVT in patients suffering from spinal infections.

This research attempts to gather routine blood and biochemical examination findings at the time of patient admission by means of a retrospective analysis conducted in two centres. The objective is to build a predictive model that will allow for early preventive intervention in patients with intertrochanteric femur fractures in the event of deep vein thrombosis.

## Materials and methods

### Section on patients

This study is a retrospective cohort one that was carried out at two sizable Chinese medical facilities. Between January 2017 and January 2022, patients with intertrochanteric femur fractures were the subjects of the study. The following were the inclusion criteria: Patients who meet two criteria: (1) have intertrochanteric femur fractures; (2) are older than eighteen years of age; and (3) exhibit discontinuity or significant displacement of the cortical bone between the intertrochanteric region on X-ray or computed tomography scans, as well as significant clinical symptoms in the hip following trauma. (1) Inability to obtain vascular colour Doppler ultrasonography results; (2) Multiple fractures or pathological fractures diagnosed; (3) Recent use of anticoagulant or antiplatelet medicines; (4) Immunological system or haematological abnormalities present were the exclusion criteria. The training group consisted of eligible patients from Guangxi Medical University's First Affiliated Hospital, whereas the external validation group consisted of eligible patients from Huizhou Central People's Hospital.

### Gathering and defining data

The patients' baseline clinical information, laboratory test results, and blood biochemistry, including coagulation function and complete blood count, were gathered for this study. Gender, age, the side that was afflicted, a history of diabetes, hypertension, alcohol use, and smoking were all included in the baseline clinical data. To confirm that the data from the two centres are comparable, we will compare their baseline data.

Furthermore, upon admission, laboratory test results were obtained from the patients for this study, which included the following information: aspartate aminotransferases (AST), alanine aminotransferases (ALT), albumin (ALB), white blood cells (WBC), red blood cells (RBC), haemoglobin (HGB), platelets (PLT), mean corpuscular volume (MCV), neutrophil (NE), lymphocytes (LYM), high-density lipoprotein cholesterol (HDL-C), creatinine (Cr), potassium (K), sodium (Na), chloride (Cl), magnesium (Mg), prothrombin time (PT), activated partial thromboplastin time (APTT).

### Outcome

The end event in this trial will be the development of DVT in patients with IFF prior to surgery. Prior to surgery, every patient had a thorough vascular colour Doppler ultrasound. The presence or absence of DVT will be determined by comparing the positive result, which indicates the existence of DVT, with the negative result, which indicates the lack of DVT.

### Analysis of statistics

Using SPSS 21.0 (SPSS Inc., Chicago, IL), the baseline data from the training and validation groups were initially compared in this investigation. After combining the data from the training and validation groups, the groups were contrasted according to whether DVT was present or absent. A range of laboratory and clinical data were compared between the groups.

The Shapiro–Wilk test was employed to evaluate the normality of continuous data. When presenting data that had a normal distribution, the mean ± standard deviation was utilised, and group comparisons were made using independent one-way analysis of variance. The Kruskal–Wallis test was used for group comparisons, and the results were reported as median (25th percentile, 75th percentile) if they did not follow a normal distribution.

Frequencies (percentages) were used to characterise the categorical data, and the Chi-square test or Fisher's exact test was used to compare groups of data. A p-value that was less than 0.05 on both sides was deemed statistically significant.

We can use GraphPad Prism 9.5.0 to plot the receiver operating characteristic curve (ROC) for factors with substantial differences. The clinical factors can be categorised using the best cutoff value. To assess independent predictors of DVT occurrence, do univariate logistic regression analysis and incorporate covariates with *p* value < 0.1 in the multivariate logistic regression analysis.

R Studio (version 4.2.2) can be used to generate the nomogram for independent predictors. Use the area under the curve (AUC) and the ROC curve to assess the model's predictive ability. Utilising the calibration plot, determine the model's average error. Examine the model's clinical benefit with a decision curve analysis (DCA) plot.

A two-sided *p*-value of less than 0.05 is deemed significant for all tests.

## Result

**Fig. 1:**
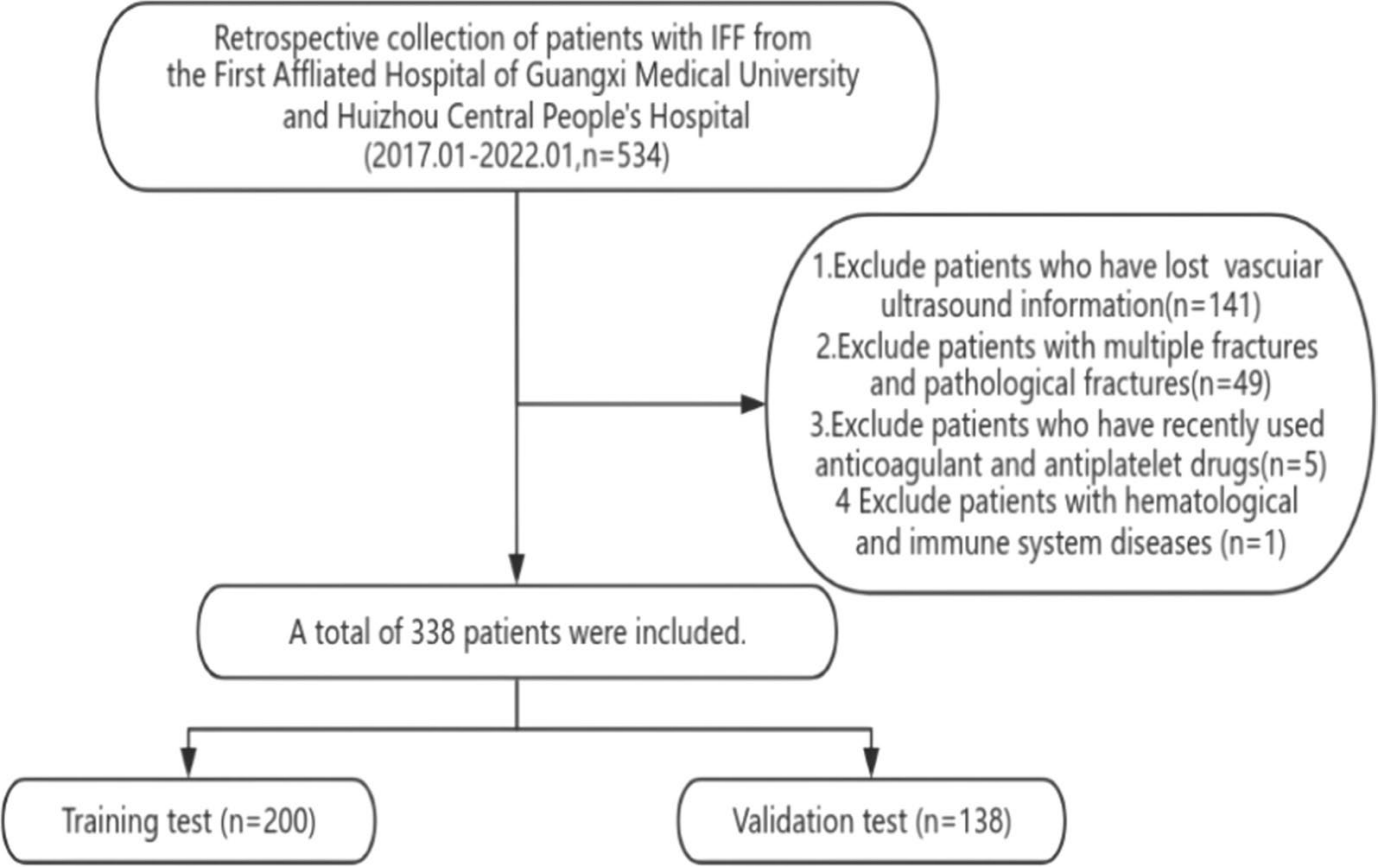
Flow chart of this study

Five hundred and thirty-four individuals with intertrochanteric femur fractures were gathered for this investigation from two sizable medical facilities. Figure 1 illustrates that 338 individuals in all were included in the retrospective analysis, but 196 patients were excluded for a variety of reasons. There were 200 patients in the training group and 138 patients in the validation group. To confirm that there was no selection bias in this investigation, we used intergroup comparison to show that there was no statistically significant difference in baseline data between the patient groups that were included and those that were excluded (Table 1).

**Table 1:** Baseline data table for comparison

Variable	Included group (n = 338)	Excluded group (n = 196)	*p*	Training test (n = 200)	Validation test (n = 138)	*p*
Sex			0.670			0.744
Male	153 (45.266%)	85 (43.367%)		92 (46%)	61 (44.2%)	
Female	185 (54.734%)	111 (56.633%)		108 (54%)	77 (55.8%)	
Age	80.5 (69,86)	81 (70,85)	0.657	79.5 (66,86)	81.5 (72,85)	0.123
Side			0.501			0.703
Left	183 (54.142%)	112 (57.143%)		110 (55%)	73 (52.9%)	
Right	155 (45.858%)	84 (42.857%)		90 (45%)	65 (47.1%)	
Hypertension			0.439			0.881
Yes	160 (47.337%)	86 (43.878%)		94 (47%)	66 (47.83%)	
No	178 (52.663%)	110 (56.122%)		106 (53%)	72 (52.17%)	
Diabetes			0.653			0.874
Yes	133 (39.349%)	81 (41.327%)		78 (39%)	55 (39.86%)	
No	205 (60.651%)	115 (58.673%)		122 (61%)	83 (60.14%)	
Smoke			0.244			0.474
Yes	145 (42.899%)	74 (37.755%)		89 (44.5%)	56 (40.58%)	
No	193 (57.101%)	122 (62.245%)		111 (55.5%)	82 (59.42%)	
Alcoholism			0.192			0.858
Yes	67 (19.822%)	30 (15.306%)		39 (19.5%)	28 (20.29%)	
No	271 (80.178%)	166 (84.694%)		161 (80.5%)	110 (79.71%)	

The two groups' baseline data were compared. With a median age of 79.5 years (66, 86), there were 92 female patients (46%), making up the training group. 110 patients (or 55%) had fractures to their left intertrochanteric femur. There were 94 (47%), 78 (39%), 89 (44.5%), and 39 (19.5%) cases of hypertension, diabetes, smoking history, and alcohol use history, respectively. The comparability of the data was demonstrated by the lack of statistically significant variations (*p* > 0.05) in the baseline data between the two groups (Table 1).

**Fig. 2:**
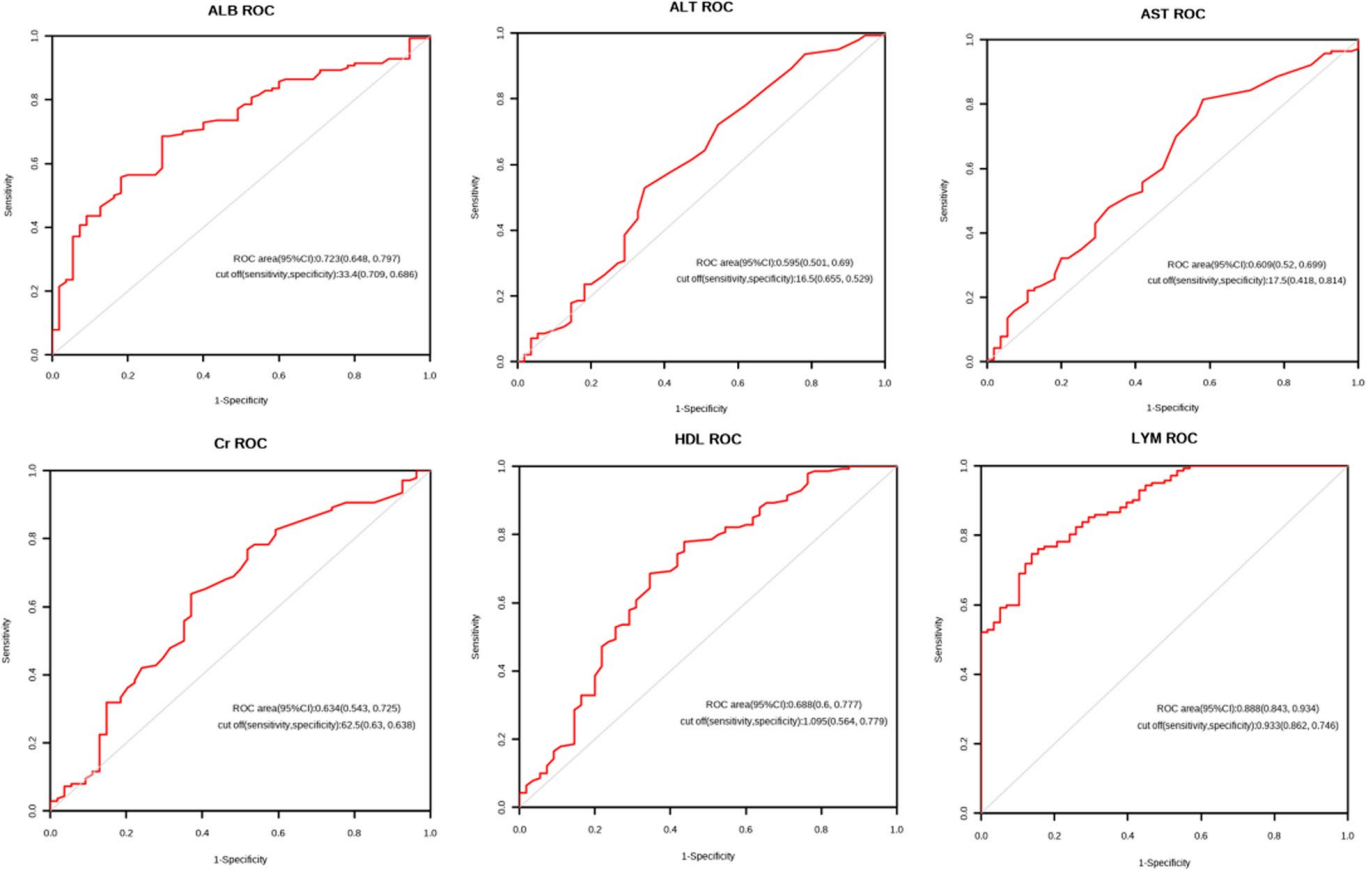
ROC curves for statistically different factors

Based on whether DVT was present or not, the data from the two groups were merged and split into the DVT group and the No DVT group. Table 2 displays significantly significant differences (*p* < 0.05) in LYM, HDL-C, ALB, AST, ALT, and Cr between the two groups. An ROC curve was created using the parameters that had substantial differences, and the area under the curve (AUC) was computed (Fig. 2).

**Table 2:** Comparison of clinical factors between the DVT group and the No DVT group

Variable	No DVT group (n = 142)	DVT group (n = 58)	*p*
Sex			0.076
Male	71 (50%)	21 (36.21%)	
Female	71 (50%)	37 (63.79%)	
Year	79 (64,86)	80 (69,86)	0.598
Side			0.975
Left	78 (54.93%)	32 (55.17%)	
Right	64 (45.07%)	26 (44.83%)	
Hypertension			0.817
Yes	66 (46.48%)	28 (48.28%)	
No	76 (53.52%)	30 (51.72%)	
Diabetes			0.403
Yes	58 (40.85%)	20 (34.48%)	
No	84 (59.15%)	38 (65.52%)	
Smoke			0.068
Yes	69 (48.59%)	20 (34.48%)	
No	73 (51.41%)	38 (65.52%)	
Alcoholism			0.290
Yes	25 (17.61%)	14 (24.14%)	
No	117 (82.39%)	44 (75.86%)	
WBC	8.93 (7,11.72)	8.89 (7.45,10.72)	0.741
RBC	3.78 ± 0.86	3.84 ± 0.87	0.650
HGB	106.26 ± 24.13	107.94 ± 18.98	0.601
PLT	221.8 (182.93,295.75)	217.6 (183.67,272.25)	0.810
MCV	89.2 (82.25,93.21)	89.68 (84.45,93.37)	0.676
NE	6.8 (4.99,9.14)	7.09 (5.14,8.88)	0.966
HDL-C	1.35 (1.11,1.65)	1.09 (0.9,1.35)	< 0.001
LYM	1.28 (0.93,1.69)	0.48 (0.13,0.81)	< 0.001
ALB	35.49 ± 5.17	31.77 ± 4.04	< 0.001
AST	25 (19,35)	20 (16,29.5)	0.017
ALT	17 (12,25.25)	14 (9.5,24)	0.038
TBIL	12.5 (8.47,18.15)	14.6 (10.1,20.25)	0.171
Cr	65.5 (57,82.75)	58.5 (48.5,69)	0.004
K	3.95 ± 0.54	3.93 ± 0.58	0.882
Na	138.65 (136.3,140.93)	139.2 (137.1,140.65)	0.501
Cl	104.25 (101.57,106.62)	105.3 (102.85,106.95)	0.152
Mg	0.86 ± 0.11	0.83 ± 0.1	0.116
PT	11.95 (11.33,12.7)	11.95 (11.25,12.6)	0.677
APTT	30.1 (28.5,31.85)	30.4 (28.92,32.25)	0.735

Univariate logistic regression analysis was performed with clinical factors included. The results indicated that the following factors were predictive of DVT: HDL-C (0.22 [0.11–0.43], *p* < 0.001), LYM (0.05 [0.02, 0.13], *p* < 0.001), ALB (0.19 [0.1, 0.13], *p* < 0.001), AST (0.32 [0.16, 0.63], *p* = 0.001), ALT (0.47 [0.25–0.9], *p* = 0.023), Cr (0.33 [0.17–0.64], *p* = 0.001), and Cl (2.53 [1.14–5.62], *p* = 0.023) (Table 3). The multivariate logistic regression analysis incorporated the significant factors found in the univariate study (*p* < 0.1). Table 3 displays the data, which indicate that ALB (0.83[0.74, 0.94], *p* = 0.002), LYM (0.02 [0.01–0.09], *p* < 0.001), and HDL-C (0.18[0.04, 0.71], *p* = 0.014) are independent predictors.

**Table 3:** Single and multivariate binary logistic regression analysis results

Variable	OR [95% CI]	*p*	OR [95% CI]	*p*
Sex		0.077		0.872
Male	1		1	
Female	1.76[0.94,3.3]		0.57[0,559.71]	
Year		0.185		
≤ 64.5	1			
> 64.5	1.69[0.78,3.68]			
Side		0.975		
Left	1			
Right	0.99[0.54,1.83]			
Hypertension		0.817		
Yes	1			
No	0.93[0.5,1.72]			
Diabetes		0.403		
Yes	1			
No	1.31[0.69,2.48]			
Smoke		0.070		0.868
Yes	1		1	
No	1.8[0.95,3.38]		1.79[0,1749.02]	
Alcoholism		0.292		
Yes	1			
No	0.67[0.32,1.41]			
WBC		0.105		
≤ 11.125	1			
> 11.125	0.54[0.26,1.14]			
RBC		0.225		
≤ 2.53	1			
> 2.53	0.47[0.14,1.6]			
HGB		0.115		
≤ 94.2	1			
> 94.2	1.77[0.87,3.59]			
PLT		0.060		0.558
≤ 333.85	1		1	
> 333.85	0.35[0.11,1.05]		1[0.99,1]	
MCV		0.157		
≤ 88.465	1			
> 88.465	1.57[0.84,2.95]			
NE		0.430		
≤ 6.12017	1			
> 6.12017	1.29[0.69,2.42]			
HDL-C		< 0.001		0.014
≤ 1.095	1		1	
> 1.095	0.22[0.11, 0.43]		0.18[0.04,0.71]	
LYM		< 0.001		< 0.001
≤ 0.93327	1		1	
> 0.93327	0.05[0.02,0.13]		0.02[0.01,0.09]	
ALB		< 0.001		0.002
≤ 33.4	1		1	
> 33.4	0.19[0.1, 0.37]		0.83[0.74,0.94]	
AST		0.001		0.099
> 17.5	1		1	
≤ 17.5	0.32[0.16, 0.63]		0.96[0.91,1.01]	
ALT		0.023		0.490
> 16.5	1		1	
≤ 16.5	0.47[0.25, 0.9]		1.02[0.97,1.06]	
TBIL		0.055		0.433
≤ 13.85	1		1	
> 13.85	1.86[0.99, 3.49]		0.99[0.97,1.01]	
Cr		0.001		0.219
> 62.5	1		1	
≤ 62.5	0.33[0.17, 0.64]		0.99[0.98,1]	
K		0.115		
≤ 3.955	1			
> 3.955	0.48[0.19, 1.2]			
Na		0.206		
≤ 139.15	1			
> 139.15	1.5[0.8, 2.81]			
Cl		0.023		0.199
≤ 102.25	1		1	
> 102.25	2.53[1.14, 5.62]		1.07[0.97,1.18]	
Mg		0.944		
≤ 0.855	1			
> 0.855	1.02[0.55, 1.91]			
PT		0.739		
≤ 12.25	1			
> 12.25	1.17[0.47, 2.92]			
APTT		0.331		
≤ 29.85	1			
> 29.85	1.59[0.62, 4.05]			

**Fig. 3:**
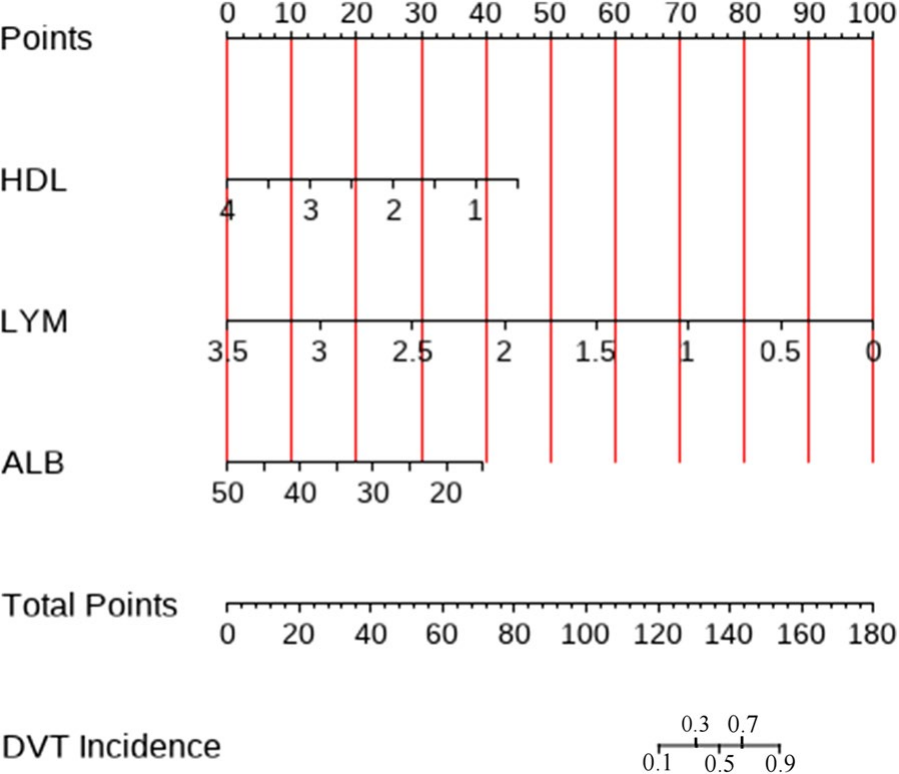
The nomogram of the study

**Fig. 4:**
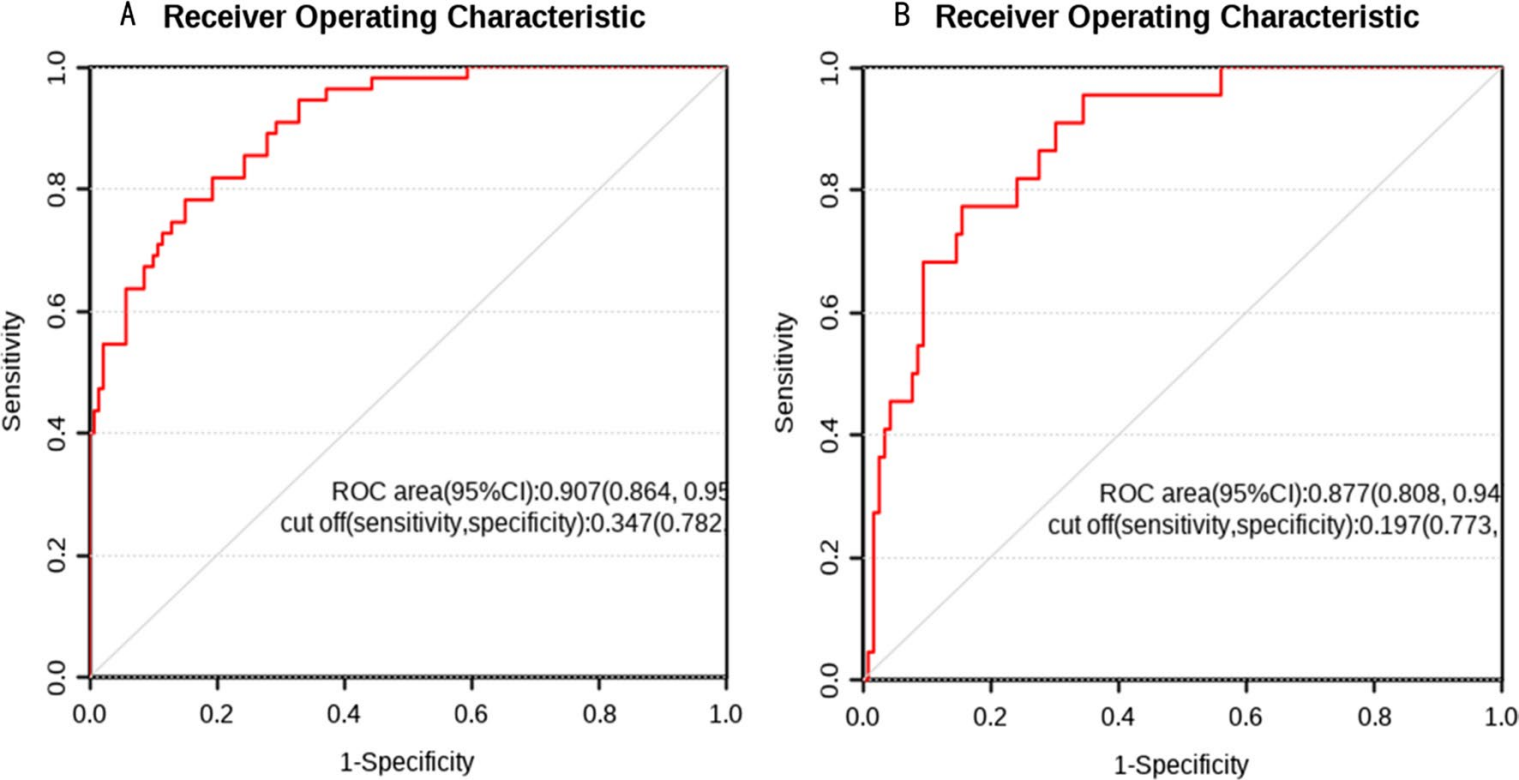
**A** ROC curve of training test. **B** ROC curve of validation test

**Fig. 5:**
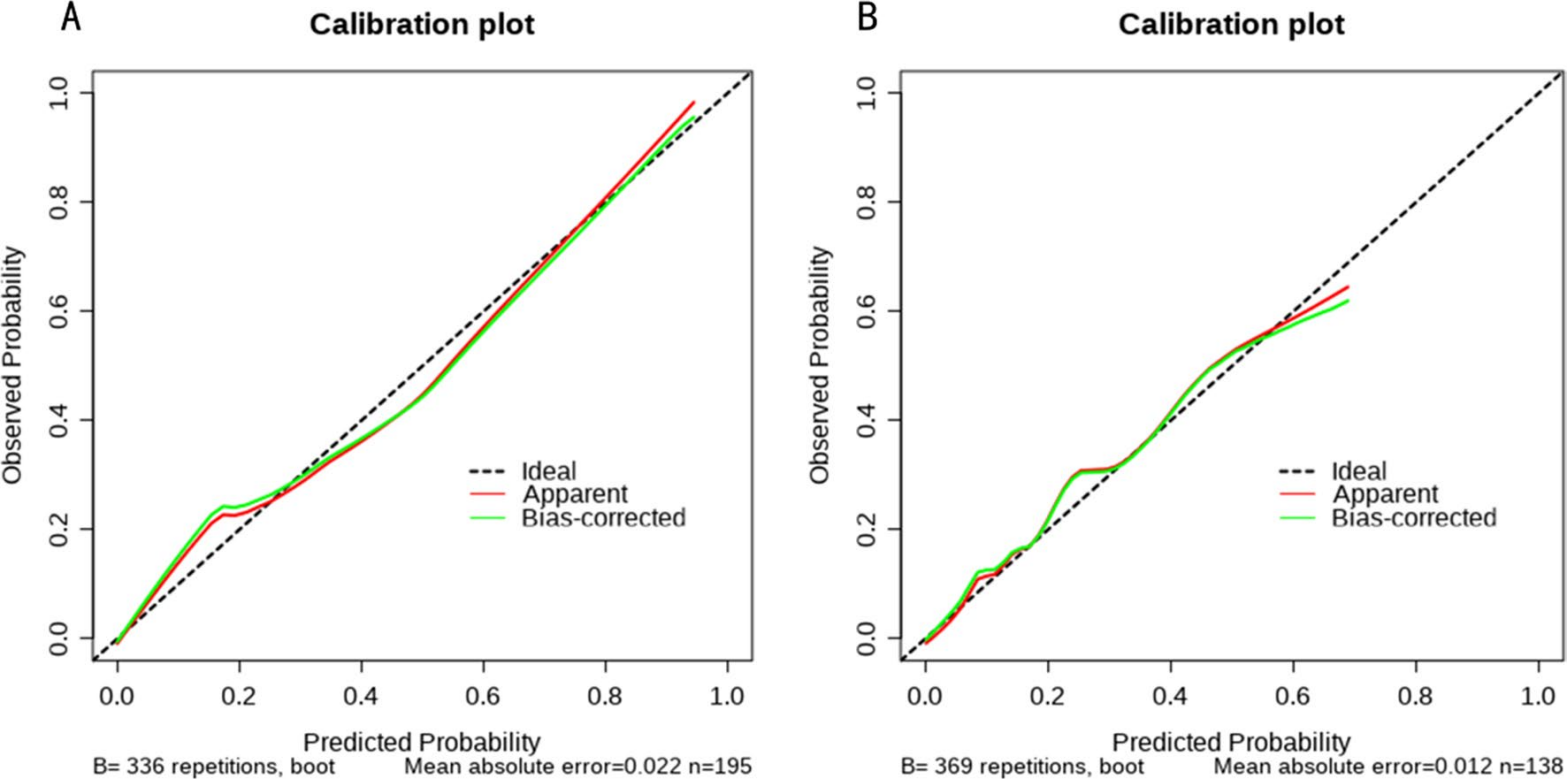
**A** Calibration curve for training test. **B** Calibration curve for validation test

**Fig. 6:**
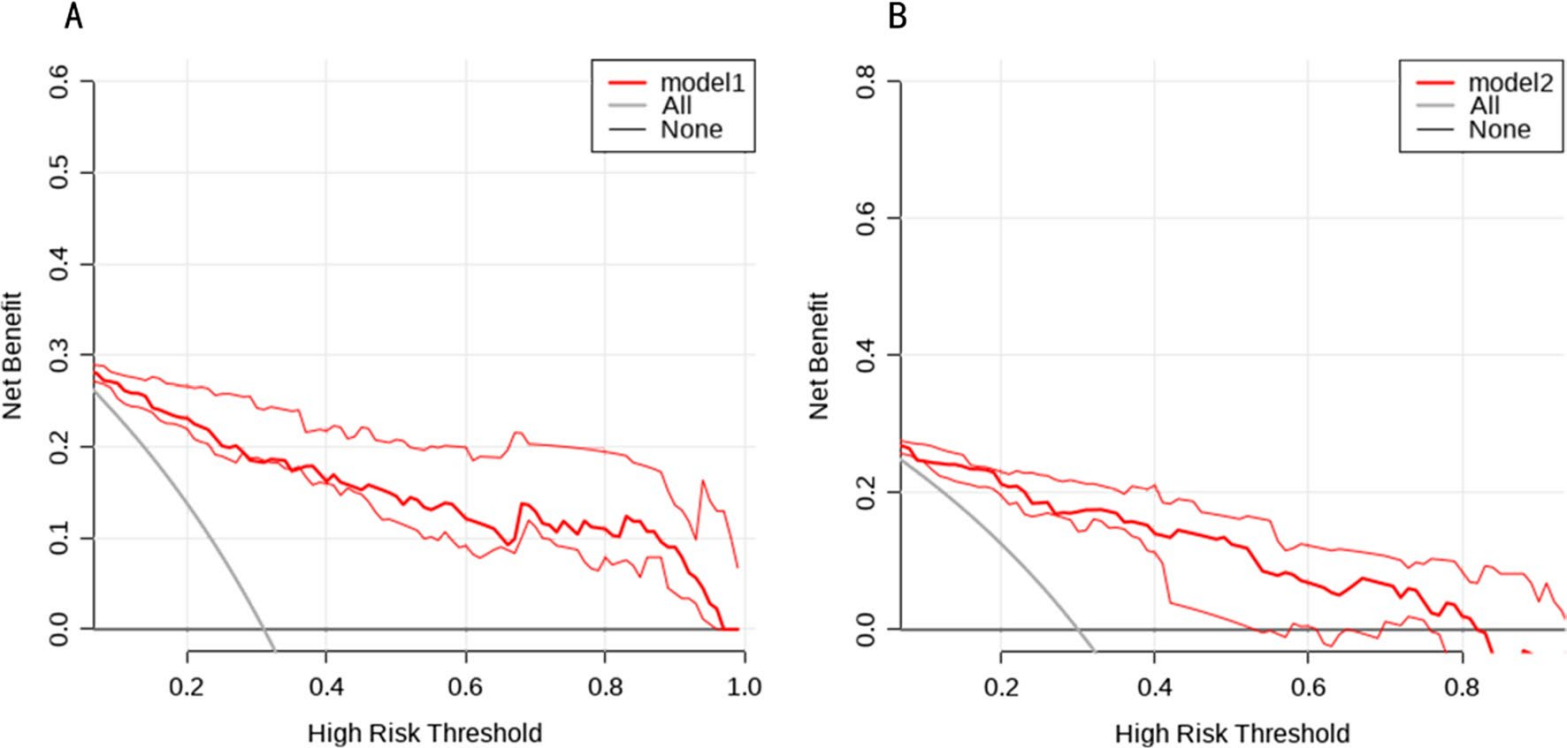
**A** DCA curve for training test. **B** DCA curve for validation test

Using R Studio software, we created a nomogram model based on multifactor analysis to further test the prediction power of several factors on patients with IFF. We created a nomogram (Fig. 3) using the training group data. Plotting the training group's ROC curve (Fig. 4A), which shows an excellent predictive ability with a C-index of 0.907, verified the nomogram's predictive performance. The training group's calibration curve (Fig. 5A) revealed an average inaccuracy of 0.022. In the threshold range of 0.01–0.95, the training group's decision curve analysis (DCA) (Fig. 6A) showed good clinical benefit.

The data from the validation group were used for validation at the same time. The nomogram's (Fig. 4B) ROC curve showed a C-index of 0.877. An average inaccuracy of 0.012 was displayed by the calibration curve (Fig. 5B). Good clinical effect was shown by the DCA (Fig. 6B) within the threshold range of 0.01–0.85.

To sum up, the nomogram performed well in terms of prediction with few mistakes in both the training and validation groups, which was advantageous for most clinical patients.

## Discussion

Preoperative deep vein thrombosis (DVT) is more common in female, elderly, long-term hospital patients, smokers, and patients with underlying medical conditions such liver and kidney diseases, as evidenced by the previous meta-analysis of DVT in patients with hip fractures [22, 23]. In order to reduce the influence of confounding factors, we therefore thought about incorporating these components in the study prior to performing a multivariate regression analysis. A nomogram model was created based on the findings, which demonstrated that lymphocytes, albumin, and high-density lipoprotein cholesterol are independent predictors of preoperative DVT in patients with IFF.

The effect of HDL-C on the development of deep vein thrombosis has drawn the attention of numerous academics. The primary way that HDL-C inhibits platelet aggregation is by downregulating the formation of thromboxane A2. It also inhibits platelet activation by producing and releasing bioactive molecules. Furthermore, HDL-C has been shown to support endothelial cell integrity [24, 25]. Through a retrospective analysis, Xu Bin-Bin et al. [26] confirmed the independent connection between low plasma HDL-C levels and the development of DVT in patients who had suffered severe fractures. Patients with deep vein thrombosis had a significantly higher monocyte-to-HDL ratio, according to Doğan et al. [27]. Moreover, researchers have looked into how serum albumin affects the development of deep vein thrombosis. According to Wu Yi-Lun et al. [28], serum albumin can be utilised as a predictor of DVT risk and is linked to preoperative DVT in older individuals with hip fractures. Serum albumin levels may be able to predict venous thromboembolism in rheumatology inpatients, according to Peng et al. [29]. Furthermore, lymphocytes, one type of inflammatory agent, also play a role in the development of deep vein thrombosis. According to research by Hasselwander Solveig et al. [30], mice devoid of B lymphocytes indirectly encourage the development of venous thrombosis. Furthermore, it has been demonstrated that a multitude of inflammatory variables are linked to the development of deep vein thrombosis [31].

Finding out a patient's DVT incidence rate as soon as possible is essential since DVT therapy and prevention have a big clinical impact. As a result, the creation of nomograms for the occurrence of DVT has gained popularity among academics in recent years. Nevertheless, there isn't presently a nomogram that uses LYM, ALB, and HDL-C to forecast the risk of DVT in individuals who have intertrochanteric fractures. Constructed based on the blood test results of patients upon admission, the nomogram in this study offers strong prediction performance and can be clinically beneficial for a large number of patients.

There are several benefits to this study: (1) This work is a retrospective analysis from two Chinese centres, and the model is dependable and simple to use because the nomogram has been verified by an external validation set. In order to achieve early prediction and assist more patients, the study's second goal is to gather pertinent data at the time of admission.

As of right moment, we have two study limitations to recognise. First off, our knowledge of the incidence and management of DVT is limited since we were unable to follow up with patients for long-term and postoperative DVT occurrence. This is primarily because most patients receive prophylactic anticoagulant medication following surgery, which surely affects the outcome significantly. Second, we want to increase the quantity of data and centres in the future. Even though we have already conducted our study in two centres and found no statistically significant differences in the baseline data between the excluded and included groups, we still wish to further validate our findings by extending our study to more centres in order to benefit a greater number of patients.

## Conclusion

In order to achieve the goal of early prevention and treatment and benefit more patients, clinical practitioners can evaluate the risk of DVT incidence in the early stages with the assistance of the nomogram built based on HDL-C, ALB, and LYM.

## Data Availability

The data used to support the findings of this study are available from the corresponding author upon request.

## Abbreviations

IFF: Intertrochanteric femur fracture
DVT: Deep vein thrombosis
WBC: White blood cell
RBC: Red blood cell
HGB: Haemoglobin
PLT: Platelet
MCV: Mean corpuscular volume
NE: Neutrophil count
LYM: Lymphocyte
HDL-C: High-density lipoprotein cholesterol
ALB: Albumin
AST: Aspartate aminotransferase
ALT: Alanine aminotransferase
TBIL: Total bilirubin
Cr: Creatinine
K: Potassium
Na: Sodium
Cl: Chloride
Mg: Magnesium
PT: Prothrombin time
APTT: Activated partial thrombin time
OR: Odds ratio
CI: Confidence interval
AUC: The area under the curve
DCA: Decision curve analysis
ROC: Receiver operating characteristic
